# Development and Testing of a Personalized Web-Based Diet and Physical Activity Intervention Based on Motivational Interviewing and the Self-Determination Theory: Protocol for the MyLifestyleCoach Randomized Controlled Trial

**DOI:** 10.2196/14491

**Published:** 2020-02-04

**Authors:** Juul M J Coumans, Catherine A W Bolman, Stijn A H Friederichs, Anke Oenema, Lilian Lechner

**Affiliations:** 1 Department of Health Psychology Faculty of Psychology Open University of the Netherlands Heerlen Netherlands; 2 Department of Clinical Psychology Faculty of Psychology Open University of the Netherlands Heerlen Netherlands; 3 Department of Health Promotion Caphri, Faculty of Health, Medicine and Life Sciences Maastricht University Maastricht Netherlands

**Keywords:** diet, physical activity, eHealth, computer-tailoring, intervention mapping, self-determination theory, motivational interviewing

## Abstract

**Background:**

Unhealthy dietary patterns and insufficient physical activity (PA) are associated with negative health outcomes, such as cardiovascular diseases, type 2 diabetes, cancer, overweight, and obesity. This makes the promotion of healthy dietary and PA behaviors a public health priority.

**Objective:**

This paper describes the development, design, and evaluation protocol of a Web-based computer-tailored (CT) dietary and PA promotion intervention, *MyLifestyleCoach*. A Web-based format was chosen for its accessibility and large-scale reach and low-cost potential. To achieve effective and persistent behavioral change, this innovative intervention is tailored to individual characteristics and is based on the self-determination theory and motivational interviewing (MI).

**Methods:**

The 6 steps of the intervention mapping protocol were used to systematically develop *MyLifestyleCoach* based on the existing effective CT PA promotion intervention *I Move*. The *MyLifestyleCoach* intervention consists of 2 modules: *I Move*, which is aimed at promoting PA, and *I Eat*, which is aimed at promoting healthy eating. Development of the *I Eat* module was informed by the previously developed *I Move*. Both modules were integrated to form the comprehensive *MyLifestyleCoach* program. Furthermore, *I Move* was slightly adapted, for example, the new Dutch PA guidelines were implemented. A randomized controlled trial consisting of an intervention condition and waiting list control group will be used to evaluate the effectiveness of the intervention on diet and PA.

**Results:**

Self-reported measures take place at baseline, 6 months, and 12 months after baseline. Enrollment started in October 2018 and will be completed in June 2020. Data analysis is currently under way, and the first results are expected to be submitted for publication in 2020.

**Conclusions:**

*MyLifestyleCoach* is one of the first interventions to translate and apply self-determination theory and techniques from MI in Web-based computer tailoring for an intervention targeting PA and dietary behavior. Intervention mapping served as a blueprint for the development of this intervention. We will evaluate whether this approach is also successful in promoting eating healthier and increasing PA using an randomized controlled trial by comparing the intervention to a waiting list control condition. The results will provide an insight into the short- and long-term efficacy and will result in recommendations for the implementation and promotion of healthy eating and PA among adults in the Netherlands.

**Trial Registration:**

Dutch Trial Register NL7333; https://www.trialregister.nl/trial/7333

**International Registered Report Identifier (IRRID):**

DERR1-10.2196/14491

## Introduction

### Background

Unhealthy lifestyle behaviors such as insufficient physical activity (PA) and unhealthy nutrition increase the risk of developing a variety of diseases, such as cardiovascular disease, type 2 diabetes, osteoporosis, cancer, and depression [1-3]. The Dutch PA guidelines state that adults should accumulate at least 2.5 hours of moderate-intensity PA every week and carry out muscle- and bone-strengthening activities at least twice a week. Daily sedentary behavior should be limited [4]. The Dutch guidelines on diet state that adults should consume at least 200 g of fruit and 250 g of vegetables daily, eat fish at least once a week, and limit intakes of energy and saturated fat by, for example, consuming fewer snacks [5,6]. Unfortunately, many adults in the world, including Dutch adults, do not follow these recommendations (eg, 50% of the Dutch adults do not meet the PA guidelines and Dutch adults consume only about 120 g of vegetables per day), and the proportion who fail to do so is higher in groups with low socioeconomic status (SES) [7-9]. As a consequence, promotion of PA and healthy dietary intake is of great importance to public health.

Effective interventions with a large reach (ie, reaching the entire population at risk because of an unhealthy lifestyle) are needed to achieve improvements in lifestyle at population level. If an intervention is to be effective and produce long-term, sustainable behavioral changes, it is important that people are able to choose their own goals and modules, that is, have a degree of autonomy within the context of the intervention, as this improves engagement and motivation for behavioral change [10,11]. A PA intervention that meets these requirements has already been developed and evaluated, and it is called *I Move*. *I Move* was successful in increasing PA levels in Dutch adults [12-14]. It is a computer-tailored (CT) intervention that can provide a large number of people with individualized feedback, taking their choices into account, at a relatively low cost. *I Move* is based on the self-determination theory (SDT) and uses the communication techniques of motivational interviewing (MI) to guide participants toward behavioral change [12]. The general idea is that by supporting the basic psychological needs for autonomy, competence, and relatedness, individuals are stimulated to develop more autonomous forms of motivation toward adoption and maintenance of targeted behavior [15]. More detailed information regarding the theoretical framework of SDT and the practical application of MI is provided in the Results section (see Step 3: Program Design). The success of this program has led to calls for the approach to be extended to other behaviors such as healthy eating, in the form a comprehensive healthy lifestyle program.

### Study Aims

We developed *MyLifestyleCoach*, a combined Web-based intervention intended to improve diet and increase PA using the intervention mapping (IM) protocol. *MyLifestyleCoach* consists of 2 modules: (1) *I Eat* (*Ik Eet*), which is intended to (increase users’ motivation to) eat healthily and (2) the pre-existing *I Move* (*Ik Beweeg*), which is intended to (increase motivation to) become more physically active. The intervention combines computer tailoring with the theoretical insights of SDT and practical applications of MI. The intervention is specifically aimed at people with a low SES. People with a low SES have the highest levels of risk behaviors and are least responsive to existing lifestyle interventions [16-18]. The purpose of this paper is to describe the development process, design, and evaluation protocol of the general program *MyLifestyleCoach* and the systematic development of the *I Eat* module (as the *I Move* program has already been developed and tested) according to the principles of SDT and MI. This insight is useful for the development of future dietary and PA interventions.

## Methods

*MyLifestyleCoach* is a Web-based, CT intervention that consists of 2 modules, *I Eat* and *I Move*, that are aimed at improving the diet and PA levels of Dutch adults, respectively. We used an adaption of the original version of I Move (see Step 4). Detailed information about the development of *I Move* can be found elsewhere [12]. *I Eat* was developed specifically for *MyLifestyleCoach* using SDT and MI, as in the development of *I Move*. The *MyLifestyleCoach* intervention was developed through the systematic adaptation and extension of *I Move*, using IM to increase the chance of producing an effective intervention [19,20]. The IM protocol consists of 6 steps, each comprising several tasks that can be used as a guide for theory and evidence-based decision-making during the design, implementation, and evaluation of a new intervention [19]. Table 1 provides a description of the steps and tasks that have to be undertaken in each step.

**Table 1:** Overview of the intervention mapping steps and the corresponding tasks.

Intervention mapping step	Task
Step 1	Needs assessment
Step 2	Program goals
Step 3	Program design (theory and practical applications)
Step 4	Program production
Step 5	Implementation plan
Step 6	Evaluation plan

In the Results section, we elaborate on these 6 steps of the IM protocol and how *I Eat* has been developed. Then, we describe the integration of these 2 modules (*I Eat* and *I Move*) in *MyLifestyleCoach*.

## Results

### Step 1: Health Problem and Needs Assessment

The *first step* of the IM protocol involves carrying out an assessment of the health problem and related behaviors. Unhealthy dietary habits, such as a low intake of fruit and vegetables and a high fat intake, carry serious health risks, for example, an increased risk of many adverse health conditions such as various types of cancer, cardiovascular diseases, and type 2 diabetes [1]. According to the World Health Organization, an adult should eat at least 400 g of fruits and vegetables a day. Less than 10% (5% for additional health benefits) of the total energy intake should come from free sugars and less than 30% from fats, preferably unsaturated fats; industrial trans fats (often found in snacks) should be avoided. Finally, one should consume less than 5 g of salt per day [21]. The Dutch recommendations for a healthy diet differ slightly and are unique in a way that they are formulated in terms of the foods rather than nutrients or food and nutrients. For example, a consumption of at least 250 g vegetables and 2 portions of fruit per day is recommended along with weekly consumption of oily fish [5,6].

Using guidelines or recommendations to prompt behavioral change is more likely to induce extrinsic types of motivation than other methods, but to achieve long-term behavioral change, it is important to satisfy the basic psychological needs in an individual to induce more autonomous forms of motivation. To this end, we conducted a pilot study to tailor *MyLifestyleCoach* to the needs of the target population and to identify behavioral targets. We asked Dutch adults (N=78) to define healthy eating using their own words and to describe what they considered a healthy diet. Most participants described healthy eating in terms of content (eg, consumption of fruit and vegetables) and approach to eating (eg, consumption of a variety of foods). Preliminary results showed that the food items mentioned most frequently in connection with healthy eating were fruit and vegetables; (limiting) sugar and fat intake were also mentioned quite frequently. We had decided that our intervention would refer to foods rather than nutrients, and so we decided to target daily consumption of energy-dense snacks. Energy-dense snacks are generally high in sugar and/or fat and could, therefore, serve as a proxy for these 2 nutrients [22]. Fish consumption was also chosen as a target outcome, as this was mentioned quite frequently in the pilot study as a way to eat (more) healthily and it was a measurable dietary outcome; in addition, according to the Dutch dietary guidelines, one should consume fish once a week.

On the basis of these results, our aim was to design *I Eat* to produce a sustained increase in the consumption of vegetables, fruit, and fish and a sustained reduction in consumption of unhealthy snacks (and to maintain these new levels) in *I Eat*. Intake of these food products would be assessed with validated questionnaires. In line with SDT, participants would decide which behaviors they wanted to change and set their own goals; thus, the intervention would meet the basic need for autonomy.

### Step 2: Program Outcomes and Objectives; Logic Model of Change

In the *second step* of IM, we defined 2 overall program goals. The first outcome was to improve the diet of adults not following a healthy diet, defined here as consumption of 250 g of vegetables and 2 portions of fruit per day, complete avoidance of unhealthy snacks, and consumption of fish once a week. The program was not designed to encourage users to achieve this outcome; instead, in line with the SDT and MI principles (see Step 3: Program design), participants would choose which dietary behaviors they want to improve. They might decide to consume more vegetables or target all 4 of the food groups mentioned in our definition of a healthy diet. The second outcome was to maintain or further improve the diet of adults who followed a healthy diet (depending on the participant’s preference—eg, a participant might decide to try to consume even more fruit). Next, we broke these goals down into smaller steps (performance objectives): to decide to eat more healthily, to improve one’s diet, and to maintain a healthy diet. Thereafter, we identified change objectives, that is, the skills individuals would need to learn to reach the performance objectives. These change objectives were formulated taking into account the basic psychological needs (autonomy, competence, and relatedness) specified in SDT and the important concepts of MI [10,23]. Examples of the change objectives in the *I Eat* module are “adults can explain why eating more healthily is important for them,” “adults are able to create an eating behavior action plan that takes into account their personal preferences,” “adults are confident that they know how to eat more healthily,” and “adults have strategies for coping with barriers to healthy eating.” See Table 2 for a selection of the change objectives.

**Table 2:** Selection of change objectives for eating more healthily.

Performance objectives		Determinants					
		Autonomy		Competence		Relatedness	
**Decide to eat more healthily**							
	Monitoring personal diet		Getting an insight into their current personal diet in an autonomous way, with little external control		Feeling confident to monitor personal diet		Feeling comfortable to think over and discuss their current diet in communication with the program
	Getting an insight into personal importance		Getting an insight into their personal importance by themselves, with little external control		None^a^		Being at ease to think over and discuss the importance of increasing their diet in the program
**Adults improve their diet**							
	Remaining aware of the importance of eating more healthily		Remaining confident to increase their diet in an autonomous way (not imposed)		Feeling confident to eat more healthily		Feeling comfortable to think over and discuss personal confidence issues in the program
	Defining clear, achievable goals with regard to improving their diet		Defining clear, achievable goals with regard to improving their diet in an autonomous way, without being coerced to do so		None^a^		Accepting help defining clear, achievable goals with regard to eating more healthily in the program
**Adults maintain their healthy diet**							
	Developing a (coping) plan about how they can best achieve their goals and how they can deal with difficult situations		Developing a plan about how they can best maintain their diet in an autonomous way, without being coerced to do so		Feeling confident to develop a plan about how they can best maintain their diet		Accepting help developing a plan about how they can best achieve their goals in the program
	Evaluating whether goals have been achieved		Evaluating whether goals have been achieved in an autonomous way, without being coerced to do so		Feeling confident to evaluate goals in an honest way		Feeling at ease to think over and discuss personally their current diet in the program

^a^No change objectives are specified for this particular determinant.

The next step in this phase was to analyze the determinants of the selected target behaviors based on SDT and MI. Although many studies have analyzed the determinants of dietary behavior (eg, see Cox et al [24]), we conducted a second pilot study to obtain a better understanding of the current beliefs of Dutch adults regarding the importance of eating more healthily and confidence in eating more healthily (2 important constructs of SDT and MI) in their own words. Overall, 66 participants were asked to respond to a Web-based questionnaire that asked them to describe, in their own words, why healthy eating is important for them and reasons why they are confident in eating more healthily, factors that are critical to behavioral change according to the principles of MI. People may have their reasons for wanting to eat more healthily yet not succeed in changing their behavior, and therefore, we also asked respondents why it was *not* important for them to eat more healthily. In addition, we asked them to describe factors that might undermine their ability to eat more healthily or make it easier to do so. The results can be found in Multimedia Appendix 1.

This information about the beliefs and perceptions of the target users and the language they used was helpful in tailoring the *MyLifestyleCoach* and its communication style, so that it met users’ needs for relatedness and autonomy. Providing suggestions about how to overcome barriers to healthy eating might increase users’ confidence in their ability to eat healthily (self-efficacy), which is an important predictor of intention to eat healthily [25]. The support suggestions given by the pilot study participants were incorporated into the *I Eat* tailoring messages.

### Step 3: Program Design

The *third step* of IM involves selecting theoretical ideas relevant to modification of target behaviors and practical ways of achieving such modification. A method is a theory-based general process that influences behavioral determinants [19]. One important determinant of sustained behavioral change, including changes in eating patterns, is autonomous motivation [26].

SDT is a macro theory of human motivation [10,23]. It focuses on the extent to which behavior is autonomous rather than controlled. Different types of motivation are placed on a continuum, ranging from amotivation to intrinsic motivation. Amotivation is the relative absence of motivation. External regulation involves performing a behavior to conform to other people’s demands (“my partner wants me to eat more healthily”). Introjected regulation is used to describe behavior performed by an individual in response to an internal pressure to avoid feeling an emotion such as shame and guilt (“if I don’t eat healthily, I feel bad”) or to obtain self-worth. Identified regulation involves engaging in a behavior because one understands and accepts its importance (eg, the studies by Deci and Ryan [27]; “eating more healthily is important for my health”). Intrinsic motivation involves engaging in an activity for the pleasure and satisfaction inherent in it (“I enjoy eating healthily”). The central distinction here is between autonomous motivation (identified regulation and intrinsic motivation) and controlled motivation (external regulation and introjected regulation). Autonomous motivation is associated with greater commitment and longer-term maintenance of behavioral changes than the other forms of motivation, and this may apply to changing to a healthier eating pattern [10,11,23,26,28]. Promoting internal motivation to eat healthily is expected to differ from promoting internal motivation to exercise. Physical activities may be performed because they are intrinsically enjoyable or because one enjoys the challenge, but people have an innate preference for palatable (unhealthy) food [29]. Therefore, it is helpful to focus on identified regulation (autonomous motivation) for eating healthily. Once people achieve this type of motivation, they may be more likely to find activities and experiences related to eating healthily, such as preparing healthy food for the family or enjoying tasteful healthy foods, to be more intrinsically motivated.

SDT postulates that providing conditions that support the basic psychological needs facilitate the development of more autonomous forms of motivation [10,11,23,30]. The basic psychological needs are need for autonomy (to engage in behavior as a matter of choice), competence (to feel competent and confident), and relatedness (to feel connected to others and understood by them) [10,11,23,31-34].

The widely adopted MI counseling style has been found to be useful in providing individuals with the change strategies they need to modify to the extent to which the basic psychological needs of the SDT are satisfied [15]. MI is defined as “a collaborative conversation style for strengthening a person’s own motivation and commitment to change” [35]. The spirit of MI is captured in 4 terms: partnership, acceptance, compassion, and evocation. The practice of MI consists of 4 recursive processes: engaging, focusing, evoking, and planning [35]. Engaging is “the process by which both parties establish a helpful connection and a working relationship,” and focusing is “the process by which you develop and maintain a specific direction in the conversation about change.” Evoking involves eliciting the client’s own motivations to change. Planning encompasses both the development of a commitment to change and the formulation of a specific plan of action. The 4 major counseling skills required for MI are asking open-ended questions, affirmation, reflective listening, and summary reflections [35]. This widely adopted counseling style has strong parallels with SDT because of its client-centered approach. It is assumed that MI generates change strategies that will fulfill the client’s basic psychological needs for competence (eg, through the use of strategies to boost confidence), autonomy (eg, because it allows clients to discover their own reasons for wanting to change), and relatedness (eg, because the interviewer is compassionate) [15,35-37].

Evidence suggests that MI is a promising way of encouraging individuals to increase their PA, although reported effect sizes (ESs) vary [30,38]. However, there is still little evidence on how effective the SDT and MI approach is in promoting healthy eating alongside an increase in PA [38], but some empirical studies have demonstrated that utilizing MI as part of a dietary modification intervention is effective: People who received MI via phone or face to face reported, for example, an increase in fruit and vegetable intake [39,40]. Furthermore, it has been demonstrated that a tailored, text-based fruit and vegetable intervention based on constructs from SDT and MI can be successful [41]. So far, it is not known whether this SDT and MI approach is also successful in promoting a healthier diet when implemented in a Web-based environment, using computer tailoring. There are differences between a Web-based environment and a face-to-face MI. In a Web-based setting, nonverbal communication is less feasible than it is in a face-to-face counseling setting. A face-to-face setting allows the interviewer to use and register social cues such as smiling or responding to very subtle expressions of motivation, which may lead to a better understanding of the client and hence more effective encouragement of behavioral change. However, Web-based, CT MI has a potentially large reach and could be a means of providing people with individualized feedback at a relatively low cost. In comparison with text-based, CT MI, Web-based, CT MI may better simulate an interactive, collaborative conversation. Other benefits of a Web-based environment over a text-based format are the instantaneous feedback and the ability to use different types of media, such as videos [42].

Thus, the SDT and MI approach also seems a promising way of improving diet. We will decide to use the skills, processes, and spirit of MI to support users’ basic psychological needs (autonomy, competence, and relatedness) and to explore and resolve ambivalence, thus increasing the chances that they will achieve behavioral change. The same skills and processes that were used in *I Move* (see [12]) were applied to the promotion of healthier eating in *I Eat*.

The 4 key concepts or *spirit* of MI (partnership, acceptance, compassion, and evocation) were implemented in the following ways. In general, participants are asked to give their opinion or reflect on statements they made earlier. They receive specific tailored feedback, and the language of the program and feedback messages is empathetic and accepting. Multimedia Appendix 2 describes in detail how we applied the MI spirit in our Web-based CT intervention.

MI comprises 4 processes. The first process is engaging and is used to establish a working relationship. We integrated a video coach into the intervention to facilitate the development of a social relationship between a user and the program. We also included several videos telling the stories of *former participants* to provide users with an opportunity to feel connected to others. The second process is focusing, which involves seeking and maintaining the direction of the conversation and consultation. Participants are informed before they enter the intervention, through advertisements and website information, that the intervention is designed to promote healthy eating and PA. The third process is evoking, in which the participant’s own motivation for change is elicited. This process is important to elicit change talk. Several methods are used: importance and confidence ruler, value clarification, looking forward toward the consequences of eating (more) healthily if one decides to do so and maintains this new behavior, and looking back to a difficult situation and identifying how the person has managed to deal with this situation and how he or she felt afterward. Regarding the rules, follow-up questions mainly focused on eliciting change talk (eg, why did you not choose a lower number). The last process is planning. This is the bridge to change and involves giving participants the opportunity to create a specific action plan that they can try out and evaluate. These 4 processes are sequential, as each process builds on the one before; however, the counselor may go back to earlier previous processes at any point during the helping relationship (ie, the process can also be recursive). Multimedia Appendix 3 provides detailed information about these MI processes and how they were implemented in our Web-based CT intervention.

Several core communication skills are applied in the intervention: open-ended questions, reflective listening, affirming, summarizing, and informing and advising. Open-ended questions are frequently used to encourage participants to come up with their own ideas. This is essential to encourage change talk, which consecutively strengthens self-determined motivation [35]. Reflective listening is implemented using a structured approach. First, the participant is asked an open-ended question, following which he or she is asked to respond to a multiple-choice question that best reflects his or her answer. We developed unique feedback messages for each combination of multiple-choice answers. Affirming was incorporated, for example, by focusing on positive behaviors and rewarding the participant for trying and by using an empathetic style in the feedback messages. Furthermore, the participants often get summaries of their previous responses. They also receive a PDF attachment after each session; this summarizes the main content of the session. Participants are given information without being judged, and sometimes, they are asked if they want more information about a certain topic, for example, dietary recommendations. Participants are also given the opportunity to receive information through several short, expert videos. Multimedia Appendix 4 describes how these core MI skills were implemented in the Web-based environment.

### Step 4: Program Production

In *step 4* of IM, the program is produced and pretested. In the sections below, we describe the specific content of the intervention, the adaptation of the program components, and the pretest.

#### Scope and Sequence

**Figure 1:**
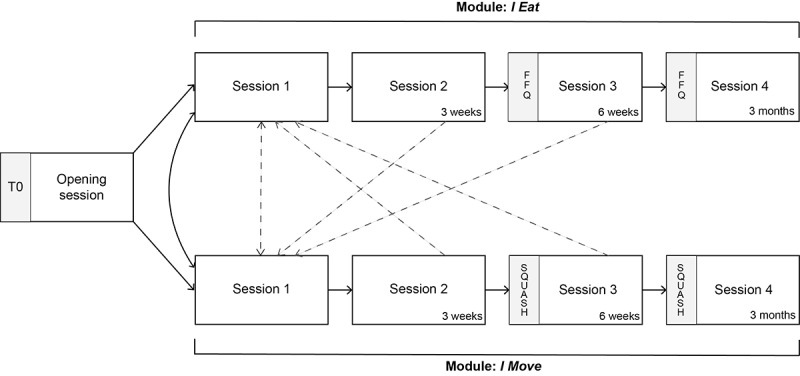
Overview of the *MyLifestyleCoach* intervention. FFQ: Food Frequency Questionnaire; SQUASH: Short QUestionnaire to ASsess Health.

**Figure 2:**
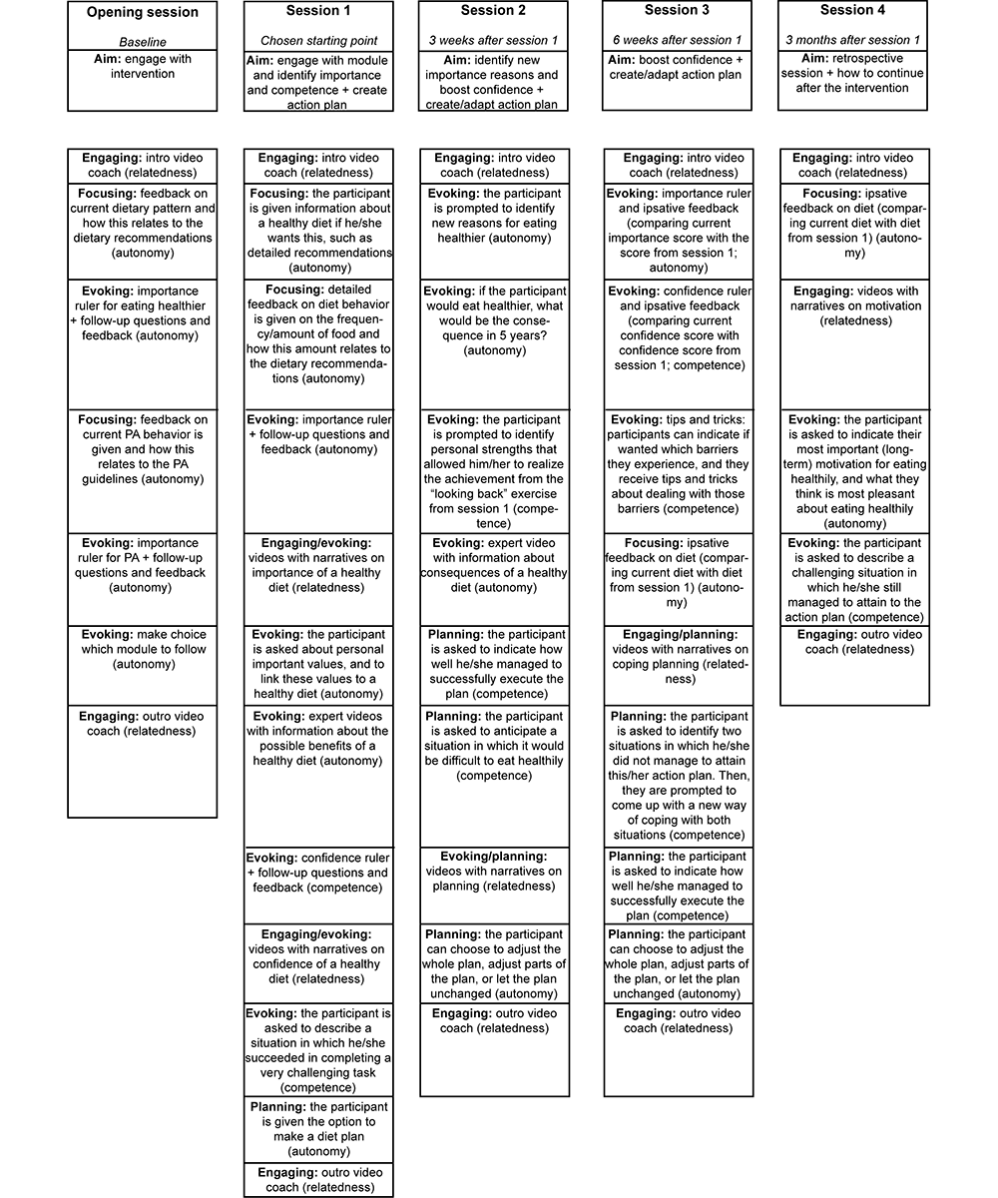
Overview of the content of the sessions of *I Eat*. PA: physical activity.

*MyLifestyleCoach* consists of an introductory session (opening session) and the modules *I Eat* and *I Move*. The program is designed in such a way that participants can choose which behavioral change module (or no module) they want to follow and if they are interested in following both modules, which module they want to visit first and when they want to use the other module. See Figure 1 for the structure of the program. Both *I Eat* and *I Move* consist of 4 sessions. Figure 2 presents the content of all the sessions (this figure has been adapted, with permission from Friederichs et al [12]).

#### Opening Session

**Figure 3:**
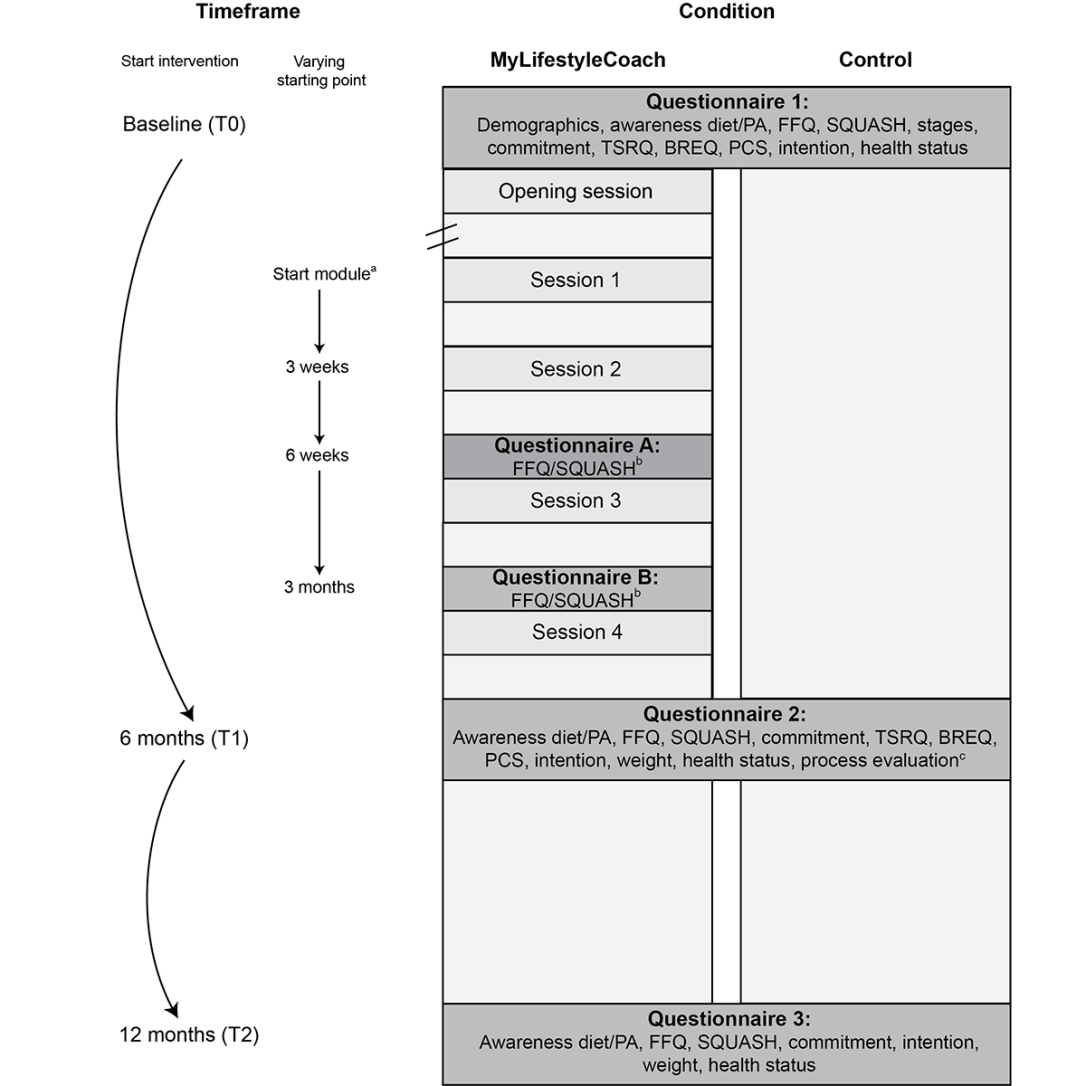
Evaluation design of MyLifestyleCoach. BREQ: Behavioral Regulation in Exercise Questionnaire; FFQ: Food Frequency Questionnaire; PA: physical activity; PCS: perceived competence scale; SQUASH: Short QUestionnaire to ASsess Health; TSRQ: Treatment Self-Regulation Questionnaire.

Before using the opening session, participants have to complete a questionnaire on several topics, including current dietary behavior and PA level, so that feedback about these behaviors can be provided. In the introduction to the opening session, participants become acquainted with the content of the program and meet the video coaches who will guide them through the program. These video coaches are used to create a sense of relatedness. Furthermore, participants receive general feedback about their current diet and PA level based on the results of the baseline questionnaire they completed beforehand. Feedback is provided using a traffic light system: The color of the light that is displayed indicates how closely their current PA level and diet correspond to health recommendations and which module (*I Eat* and/or *I Move*) is advised to use. The traffic lights are purely meant to provide participants with an insight into what they could change and are not necessarily intended to induce compliance with PA guidelines and dietary recommendations. When participants received a green traffic light advice (in line with the guidelines), they received the message that following the correspondent module was not of high priority. Nevertheless, they were free to have a look at the module. When participants received an orange traffic light advice, they received the message that following the correspondent module might be relevant to them. When participants received a red traffic light advice, they received the message that following the correspondent module might be highly relevant to them. For both the orange and red traffic light advice, they were told that they were free to have a look at the module by choosing it in the program later. Participants also have to use the importance ruler to rate how important they feel it is for them to eat more healthily and become more physically active. They are given feedback tailored to their ratings. Subsequently, participants receive a short, combined summary of their current dietary behavior and PA level, and again are given advice on which module to use. Thereafter, they have to choose whether they want to participate in *I Eat* and/or *I Move*. They are also asked whether they want to start immediately or wait (waiting is restricted to a maximum of 14 days to avoid that the follow-up questionnaires, see Figure 3 [*Start module*], and the sessions of the intervention are intertwined). Figure 3 has been adapted with permission from Friederichs et al [12]. Next, we describe the sessions of the *I Eat* module.

#### I Eat Session 1

Session 1 is the most extensive of the 4 interactive sessions. It starts with an introduction, which includes the Dutch definition of a healthy diet, and is intended to engage participants. Thereafter, participants’ awareness about their current dietary behavior is raised through the provision of detailed feedback (in terms of daily amount of vegetables, portions of fruit, consumption frequency of snacks, and weekly consumption of fish). The importance participants place on changing their behavior and their confidence in their ability to do so are critical to autonomous motivation. We assess how important they perceive healthier eating to be and how confident they are of achieving this using the importance and confidence ruler (1-10 scale) [43]. Follow-up questions are then asked based on the scores given. For example, “How important would you say it is for you to eat healthier on a scale ranging from 1 to 10?” For example, scores of less than 4 in response to the question “How important would you say it is for you to eat more healthily on a scale ranging from 1 to 10?” prompt the following: “It looks as if healthy eating is not your top priority, but perhaps there are still reasons why eating more healthily is important to you. Why could eating more healthily be important to you?” (see Multimedia Appendix 3 for more detailed information regarding these applications). Finally, intention to change (which is related to intrinsic motivation) is addressed. Users who say they intend to change are asked whether they want to make an action plan for eating more healthily. Participants are asked to formulate their goal in a concrete and realistic manner, formulate their most important motivation for eating (more) healthily, the location(s) of their choice, and on which moments they want to execute their activity. If desired, the participants can also indicate with whom they want to execute this action plan (*relatedness*) and what preparations they need to undertake before being able to implement their plan. In a pilot study of the *I Move* intervention, 85% to 90% chose to make such a plan for increasing their level of PA [44]. If the participant decides to make an action plan, the program stimulates him or her to execute it. The following sessions evaluate participants’ execution of their action plan. Participants who do not want to make a plan at this stage are given the opportunity to do so in the following sessions. At the end of this session (as well as at the end of sessions 2 and 3), participants who chose to do just the *I Eat* module are asked if they would like to start the *I Move* module as well. Those who express an interest in doing so are then asked whether they want to start immediately or select a start date up to 14 days thereafter. If they choose to start the *I Move* module immediately, they are taken to session 1 of *I Move* once they have completed their current session. If they decide to start later with *I Move,* they receive an email on the day they have selected containing a link to session 1 of the module.

#### I Eat Session 2

Three weeks after session 1, participants receive an email inviting them to session 2. Session 2 looks back at the participant’s perception of the importance of healthy eating. Participants are also asked whether they have identified new reasons to eat more healthily. In addition, participants are asked about what effects they think it would have if they started eating more healthily at this point. After that, participants are asked to think of situations in which they would find it difficult to eat healthily and to think of strategies they might use to cope with these situations. Thereafter, the participants are invited to identify personal strengths to boost their confidence. They are asked to elaborate on how their personal strengths (such as perseverance) are helpful in dealing with such difficult situations. This is a helpful strategy in the change process. Finally, the participants who made an action plan to help them eat more healthily in the previous session are given the option to evaluate how well they have succeeded in following it. They are given the opportunity to alter their plan and formulate additional coping plans. Those who did not make a plan are again given another opportunity to do so.

#### I Eat Session 3

At 6 weeks after session 1 (ie, 3 weeks after session 2), participants receive an email invitation to session 3. This session begins with assessment of participants’ current perception of the importance of healthy eating and their confidence in their ability to do so, which is carried out using the rulers. Subsequently, participants are given feedback about how their perceptions compare with their perceptions at the start of *I Eat* (session 1). After the feedback message concerning confidence, the participants are asked to think back to a difficult situation in which they struggled to eat healthily but managed to overcome their difficulties. This is supposed to boost their self-efficacy and give them more confidence in their ability to eat more healthily. They are then given some tips for eating healthily in situations where it is difficult to do so, which are derived from the pilot study. After this topic, participants receive feedback on whether they have made progress toward a healthier diet (comparison of current and preintervention diet, based on the self-assessments). Finally, participants are once again given the option of evaluating and adapting their plans and formulating coping plans. If they have not yet made a personal healthy eating action plan, they are once again given the opportunity to do so.

#### I Eat Session 4

At 3 months after session 1, participants can enter session 4. This is a session in which several topics from previous sessions are covered. Participants can choose which of several topics they would like to pursue. The available topics are feedback on their dietary behavior, long-term personal motivation and confidence, how to deal with difficult situations, and information on how to maintain their new behavior after the end of the program. Participants can opt to skip one or more topics.

#### I Eat Videos

The program gives participants the option of viewing various prerecorded videos during the sessions that are mainly text based. Previous research has shown that this type of interactive video counseling has promise as a technique for public health interventions [45-48]. These videos include a human video coach who guides the participant through the program, short videos telling the stories of 4 putative *former participants* of *I Eat* (an older nurse, a younger cleaning lady, an older production assistant, and an elementary school teacher), and videos in which a dietician talks about the positive effects of eating healthily. Giving participants the opportunity to select the videos they want to see instead of making them start automatically at a certain point in a session is in line with MI practice and with the principles of SDT, which suggest that the individuals’ need and desire for information should be assessed before it is provided. The videos were scripted, and a film producer was hired to produce them. A real dietician provided feedback on the dietician’s script. An advertisement was placed on a casting website to recruit actors for these videos. To increase the credibility of the actors appearing in the videos, several colleagues in the health psychology department were asked to choose the actors (from a preselection of actors that responded to the advertisement) who were most representative of a certain description. The videos were recorded in different settings, including a green screen, hospital, high school, and domestic setting.

#### Modification of I Move

Effectiveness and reach are key to the impact of a program. *I Move* was innovative because it was one of the first interventions to integrate SDT and MI into a Web-based, CT intervention. Although *I Move* was effective in increasing PA, it was recognized that improvements were necessary to reduce the high dropout rate (607/987 participants, 61.5%, did not complete the 12-month questionnaire) and to broaden the intervention by adding a healthy nutrition module. We fine-tuned the tailoring of the feedback messages and language used to make it more suitable for people with limited education. For example, we changed passive sentences into active sentences. Furthermore, the tailored feedback was updated, so that it was consistent with the new Dutch guidelines for PA (150 min of moderate-intensity PA per week; the original *I Move* intervention focused on the accumulation of 30 min or more of moderate-intensity PA over at least 5 days per week). Finally, we made the guidelines less explicit to encourage users to set their own targets for increasing PA level rather than simply trying to achieve the recommended level.

#### Delivery Channel

As unhealthy dietary patterns are prevalent, it is important to design and implement an intervention that can reach large numbers of people. Computer tailoring can be used to create interventions with some degree of personalization to potentially reach a large population at relatively low costs. Computer-tailoring has been defined as “the adaptation of health education materials to one specific person through a largely computerized process” [49]. It could especially be beneficial in the Netherlands, where the overwhelming majority (97%) of the population aged 12 years and above has access to the internet [50], including low SES groups [51]. There are several reasons to keep computer tailoring as a technique, similar as in *I Move*, to deliver the intervention. First, the CT intervention *I Move* has been shown to be effective in increasing PA [13,14]. Research has also indicated that Web-based, CT dietary interventions are more successful in changing people’s intake of fruit, vegetables, and fat than provision of general guidance or no information [49,52-57]. Finally, CT interventions have also been found to be effective among low SES groups [58].

*MyLifestyleCoach* was developed using the TailorBuilder software (OverNite Software Europe) [59]. This software is designed to generate tailored feedback based on algorithms. The intervention is integrated into a website that provides information about the intervention and answers to frequently asked questions [60].

#### Pretest

We will conduct 2 pretests. The first will be a 3-part paper-and-pencil pretest covering (1) the design of the website, (2) evaluation of the baseline questionnaire, and (3) the opening session (N=10). The first part will use 5-point Likert scales to evaluate several possible website designs in terms of appeal and appreciation. Participants will also be asked to suggest possible improvements. The second part will focus on clarity of the questions and instructions in the baseline questionnaire. The third part will consist of several questions about the content of the opening session, the clarity of explanation of the program structure, the clarity of traffic-light feedback about dietary and PA patterns, and feedback about the importance of eating more healthily and being more physically active. We will also ask which module(s) the participants would choose to follow and why. We will ask about several attributes of the videos, such as how easy it is to relate to the content and narrators and whether they are interesting to watch. The second and third parts of this pretest will be completed once the participant has walked through the opening session on the Web.

In the second pretest, we will test the module *I Eat* (N=16). Overall, 10 participants will test the intervention at home using their own devices. They will receive a printed questionnaire to assess the user experience, which they should fill in as they proceed through the intervention. We will also conduct a qualitative evaluation with 6 other participants and will use the think-aloud method while testing the intervention. The results and suggestions from the pretests will be used to improve and finalize the intervention for the randomized controlled trial (RCT).

### Step 5: Program Adoption and Implementation Plan

The development of an implementation plan to enable adoption, implementation, and maintenance of the program is central to Step 5 of IM. As *I Eat* is also one of the first dietary interventions to integrate MI into a Web-based, CT format, the evaluation study (ie, the assessment of its effectiveness) will focus on the adoption and implementation of *MyLifestyleCoach*.

For adoption of the program in the context of the evaluation study (ie, the intention to use it and subscription to the program), we will provide information about the program through an advertisement that will be distributed via an internet research panel to recruit the participants for this study. Facilitation of program use (for implementation and sustainability) was already taken into account in the first step of the development. Minimal human action is required to participate in the intervention. Individuals who want to participate in the intervention can register to do so on the website. After registering, they will automatically receive invitation emails when a new session is available. Furthermore, several small pilot studies and interviews have been conducted with members of the target population to identify their preferences and needs regarding the content and appearance of the intervention and the intervention has been adapted accordingly. Once we have demonstrated that the program is effective in delivering the intended outcome, that is, eating (more) healthily, we may consider further implementation possibilities, such as targeting other program outcomes in other domains of public health (eg, smoking), and the use of advertisements in mass media, social media, newsletters, or certain websites for sustainability purposes.

### Step 6: Evaluation Plan

Step 6 in IM will deal with the planning of the evaluation of the combined nutrition and PA intervention. A 2-group RCT will be conducted to evaluate the efficacy of the intervention by comparing diet and PA in an intervention group and a waiting list control group. Figure 3 shows the evaluation design. Participants will be randomized over conditions; the participants who are placed in the waiting list control group will be given the opportunity to use the intervention once the study has ended. Measurements will take place on the Web at the study website at baseline (T0) and at 6 months (T1) and 12 months (T2) after baseline. In addition, 6 weeks and 3 months after the start of a module, participants in the intervention conditions will also be asked to fill in a questionnaire assessing dietary behavior or PA, depending on the chosen module (Figures 1 and 3). Participants will be given tailored feedback on their diet and/or PA in sessions 3 and 4 based on the results of these questionnaires. At the beginning of the study, all eligible participants will be given information about the study and asked to sign a Web-based informed consent form. Data collection started in October 2018 and will be completed in June 2020.

This study has been reviewed and approved by the Committee for Ethics and Consent in Research of the Open University of the Netherlands (reference number: U2018/07266/SVW). It was judged that the study is not within the scope of the Medical Research Involving Human Subjects Act; therefore, this study did have to undergo a review by an accredited Medical Research and Ethics Committee or the Central Committee on Research Involving Human Subjects. This study is registered in the Dutch Trial Register (NL7333).

#### Participants

The inclusion criteria for the participants are age between 18 and 70 years, an adequate understanding of the Dutch language, possession of a computer or tablet with access to the internet, and no participation in the *I Move* intervention or pilot studies. Participants who are not willing to sign the informed consent will be excluded.

Given the modifications made to the original *I Move* intervention, we expect to improve the ES for PA to 0.30 at 12 months [13,14]). There are no estimates of ES in an MI-planning CT dietary intervention. A review indicates that in clinical settings, MI has produced ESs of up to 0.57 [61], but this is the first Web-based MI-planning CT intervention targeting both PA and dietary habits. It might be possible to find additive effects when participants decide to start with both modules, *I Eat* and *I Move*, so we will use a conservative estimate of the ES (0.30). We expect an ES of 0.05 in the control condition. On the basis of these data, a power calculation (ES=0.25; power=0.80) indicates that a total of at least 400 participants is needed for this study (200 per condition). On the basis of an expected dropout rate of 50%, we would need to enroll at least 800 participants. We do not expect all participants to decide to do both modules, and as we want to analyze the level of participation of the modules, we will enlarge the sample size to at least 1200 participants in total. A research panel will be used to recruit these participants. The sample will be representative of the population in terms of age and gender, but we will strive for an overrepresentation (about 50% instead of the 31% in the population [62]) of people with a low educational level, which will be used as a proxy for SES.

After passing the inclusion questions and providing informed consent, two-thirds of the participants will be randomized into the intervention condition and one-third will be randomized into the waiting list control condition. We will oversample the intervention group, as we expect that many participants will not participate in both modules; oversampling provides the option to conduct separate analyses per module. After that, participants will fill in the baseline questionnaire (dietary and PA parts) and continue with the intervention. The path they follow will depend on the choices they make.

#### Attrition Prevention

Attrition from internet-delivered health interventions is a subject of particular concern. In addition to the use of SDT and MI principles, tailoring, and personalized videos, we will implement several strategies to reduce attrition. First, the participants who complete each questionnaire will enter into a draw for 50 prizes of up to US $55 (a total prize pot of US $1100). Those who complete all sessions and questionnaires, including the follow-up questionnaires at 6 and 12 months after the baseline measurement, will enter into a draw to win 1 of 2 tablet computers [63]. Second, follow-up questionnaires at 12 months will be brief, only aiming to assess diet and PA behaviors, commitment and intention [64]. Third, as up to 10% of dropouts in a previous study could be attributed to changes of email address, we will ask participants to provide telephone numbers so that we can contact people who change their email address [65]. Provision of a telephone number will be optional, and we will ask explicitly for permission to use the number.

#### Measurements

The primary outcomes of this study are diet (ie, number of portions of fruit and daily consumption of vegetables [in g]; the consumption frequencies for fish and unhealthy snacks) and PA behavior (minutes of moderate-to-vigorous activity). Diet will be assessed with a validated Food Frequency Questionnaire (FFQ) [66]. Our FFQ only includes the items about fruit, vegetables, fish (1 question assessing the consumption frequency per week), and snack foods (unsalted nuts, dried fruits, chocolate, sweets, cookies, chips, ice cream, and savory pastries); see Coumans et al [67] to know how snacking consumption frequency was determined. We added questions assessing the size of vegetables and fruit portions based on Willems et al [68]. PA behavior is assessed using the validated self-administered Dutch Short QUestionnaire to ASsess Health [69]. Furthermore, several secondary outcomes will be measured. Motivation (amotivation, controlled motivation, and autonomous motivation) is measured using the 2 Treatment Self-Regulation Questionnaires (TSRQs): one that addresses dietary behavior and the other PA behaviors [34]. As the TSRQs do not differentiate between the specific forms of autonomous motivation, we will include 2 *intrinsic regulation* subscales from the Dutch Behavioral Regulation in Exercise Questionnaire (BREQ-2) to determine intrinsic motivation for both dietary and PA behaviors, as this is the only fully self-determined form of motivation [70]. The original BREQ-2 is used to measure intrinsic motivation for exercise (but we translated exercise to *bewegen* which means *to be physically active*); we will use an adapted version of the BREQ-2 to measure intrinsic motivation to eat healthily in which we have replaced *exercise* in all items with *eating healthily* to compare intrinsic motivation for engaging in PA with a healthy diet as closely as possible. Competence will be assessed using 2 specific Perceived Competence Scales for becoming more physically active and eating more healthily [71]. Other secondary outcomes are awareness about current dietary behavior and amount of PA, stage of change for nutrition and PA, intention [72] and commitment [73] to eating more healthily and becoming more physically active, and health status, measured using a 100-point thermometer-style visual analogue scale. For the purposes of a process evaluation, participants from the intervention group will be asked what marks (1-10) they would give to the program (ie, *MyLifestyleCoach* and the module(s) they followed) [68]. Furthermore, we will assess the extent to which the intervention supports the basic psychological needs using several items from the study by Walthouwer et al [74]: 1 item on competence, 2 items on autonomy, and 3 items on relatedness.

## Discussion

### Principal Findings

This paper describes the systematic development of *I Eat* and how it has been combined with the pre-existing *I Move* program to create a Web-based intervention *MyLifestyleCoach* aimed at promoting healthy diet and PA behavior. We also describe the protocol for the design and evaluation of *MyLifestyleCoach*. This intervention combines computer tailoring with the theoretical insights of SDT and practical techniques of MI. It is specifically aimed at people of low SES. We developed the intervention using the IM protocol [19] because a systematic approach is more likely to yield an effective intervention [20]. Following the IM protocol gave us an insight into the steps needed to develop an intervention based on the adaption of an existing intervention *I Move* [12-14].

Unhealthy diet and lack of PA carry similar health risks (eg, increased risk of developing various types of cancer, cardiovascular diseases, and type 2 diabetes [1-3]). The process of changing one’s diet might be quite different from the process of changing one’s level of PA and may be even more complex. Activities concerning PA could be more related to intrinsic motivation, as they can be pursued for being enjoyable, whereas this link with intrinsic motivation may be less clear for eating (more) healthily. Other than the obvious benefits of eating more healthily (eg, losing weight), new eating behaviors may not have much immediate added value [26]. Furthermore, people have an innate preference for unhealthy (palatable) foods that are also known to enhance mood by activating reward systems, although some may enjoy the taste of healthy food as well [29]. For these reasons, the satisfaction of the basic psychological needs may be even more critical to achieve a healthier diet. We conducted a new needs assessment using 2 Web-based pilot studies because the performance and change objectives for interventions promoting increased PA and healthier eating are different. We made a point of eliciting free-text responses to gain an insight into the language used by the target population when talking about diet, foods, and eating behavior. The results were used to formulate the performance objectives. Thereafter, we specified the change objectives, which were formulated in terms of the basic psychological needs of SDT. MI counseling techniques seem to be general rather than domain specific, making them suitable tools for working toward different program goals. This made it straightforward to implement MI techniques in *I Eat,* as they could be integrated using the same approach as in *I Move* [12]. The opening session, which is designed to interest users in eating healthily and being physically active, was built from scratch. We used *I Move* as a template for the design of *I Eat*, replicating the structure and inclusion of SDT and MI elements (eg, feedback on current behavior and the importance ruler). This was done to keep the balance between the fidelity to the original design of *I Move* (thus retaining the elements that made *I Move* effective) and the adaptations required because of the difference in domain. Nevertheless, creating the *I Eat* module was the most time-consuming part of development of the intervention. After pretesting the program, we will recruit participants using an internet-based research panel. The intervention’s effectiveness will be evaluated in an RCT (intervention vs waiting list control condition). Validated questionnaires will be used to assess diet, and the evaluation will also include questionnaires covering possible moderators, mediators, and determinants of diet and PA.

Unhealthy dietary habits and inadequate PA levels are a public health concern; therefore, many interventions that attempt to improve diet and PA levels have been developed; however, the effects have often been small and lacking in persistence [55]. This may be because traditional interventions induce extrinsic motivation instead of more autonomous forms of motivation [52,55,75]. Interventions based on SDT and MI, which recognize intrinsic motivation as a key factor in sustained behavioral change, may be a better method of achieving long-term behavioral change [10,76-79]. However, this approach is innovative and challenging in both theoretical and practical terms. Previous research has demonstrated that MI can be used outside of intensive counseling contexts, for example, by telephone [80-82]. Other studies have shown that MI principles can indeed be successfully translated to computer-based formats [13,14]. However, there is only limited evidence on whether this approach is also likely to be successful in the diet domain [41,83]. *MyLifestyleCoach* is innovative, as it is among the first attempts to apply MI techniques in a Web-based, CT intervention with objectives in 2 domains, PA and diet, that allows users to choose which domain(s) they would like to target. The effectiveness of this approach is yet to be evaluated.

### Challenges and Limitations

One challenge in the development of *MyLifestyleCoach* was translating the spirit, processes, and skills of face-to-face, counseling-style MI to a Web-based environment. One of the difficulties is that a human counselor delivering face-to-face counseling may excel at expressing empathy, providing insightful reflection, and responding to very subtle expressions of change talk. It may not be possible to implement these behaviors fully in a Web-based environment. Our aim was to mimic face-to-face counseling situation as closely as possible by using a variety of MI skills and tools (importance ruler, looking back, specific empathic noncoercive feedback messages, and a combination of open and closed questioning) that could be implemented effectively within the constraints of a Web-based environment [11].

Although this is one of the first interventions targeting diet and PA based on SDT and MI developed using the IM protocol, there are several potential limitations to be noted. The time-consuming aspect of developing an intervention following the IM protocol is risky, especially in the field of electronic health. Owing to rapid technology developments, it could be that Web-based interventions may already be outdated once they have proven to be effective. Nonetheless, it is a valuable tool to develop or adapt (existing) interventions. The program is especially developed and tested in people who have the necessary digital skills. The risk could be that this study will take place in a select sample, that is, as people with a low SES or older people have highest levels of risk behaviors and are least responsive to existing lifestyle interventions, they may be less likely to be targeted [16-18]. Another limitation is that we have not addressed (yet) how the intervention will be spread to the targeted population once this intervention is shown to be effective. Another limitation is that we are recruiting our participants via a research panel. We have not yet addressed how the intervention will be spread to the targeted population once this intervention is shown to be effective.

### Conclusions

This paper describes the development of a Web-based CT intervention *MyLifestyleCoach* that is intended to motivate Dutch adults to eat more healthily and to become more physically active. We hope that by inducing more autonomous forms of motivation than traditional interventions, *MyLifestyleCoach* will elicit sustained changes in diet and PA. This is one of the first attempts to integrate SDT and MI into a Web-based, CT intervention addressing both diet and PA. Results from the RCT will provide an insight into the efficacy of the approach and could be used in the development and optimization of future Web-based interventions in several public health domains.

## Appendix

Multimedia Appendix 1 Overview of the beliefs for (no) importance, (no) confidence, support, and difficult situations toward eating healthier.

Multimedia Appendix 2 Translation of the motivational interviewing spirit into our Web-based computer-tailored intervention.

Multimedia Appendix 3 Translation of motivational interviewing processes into our Web-based computer-tailored intervention.

Multimedia Appendix 4 Translation of core motivational interviewing skills into our Web-based computer-tailored intervention.

## Abbreviations

BREQ: Behavioral Regulation in Exercise Questionnaire
CT: computer-tailored
ES: effect size
FFQ: Food Frequency Questionnaire
IM: intervention mapping
MI: motivational interviewing
PA: physical activity
RCT: randomized controlled trial
SDT: self-determination theory
SES: socioeconomic status
TSRQ: Treatment Self-Regulation Questionnaire
