# Supplementary material for: Development and Testing of a Personalized Web-Based Diet and Physical Activity Intervention Based on Motivational Interviewing and the Self-Determination Theory: Protocol for the MyLifestyleCoach Randomized Controlled Trial
Source: JMIR Res Protoc. 2020 Feb 4;9(2):e14491. doi: 10.2196/14491 (PMC7055747; doi:10.2196/14491)
Supplement: Multimedia Appendix 1 [file resprot_v9i2e14491_app1.docx]

**Multimedia Appendix 1. Overview of the beliefs for (no) importance, (no) confidence, support, and difficult situations toward eating healthier.**

| Importance | No importance | Confidence | No confidence | Support | Difficult situations |
| --- | --- | --- | --- | --- | --- |
| Health | Taste of unhealthy food is better | Motivation/goal | Temptations of unhealthy food | Availability | Parties/visit |
| Energy | Snacking should be possible | Self-efficacy | No skill/knowledge | Planning/goals | Work/school |
| Ageing | Price (too high for healthy products or unhealthy products are cheaper) | Taste of healthy food | Price (too high for healthy products or unhealthy products are cheaper) | Easy recipes | Price (too high for healthy products or unhealthy products are cheaper) |
| Weight concern | Already eating healthily | Succeeded before | Mental reasons | Having a goal (motivation) | No energy/lazy |
| Mental reasons | Availability (healthy products are less available than unhealthy products) | Availability of healthy products | Availability (healthy products are less available than unhealthy products) | Reward when eating healthier | Availability (healthy products are less available than unhealthy products) |
| — | Social reasons | Social environment | Self-efficacy issues | Avoid difficult situations | No time |
| — | No time | Habit | Social environment | Price | Being outdoor (eg, dining out) |
| — | Too much effort | Feels good | No added value | Social support | Holiday |
| — | Self-efficacy issues | Planning ability | Already eating healthy | Time | Stress/emotions |
| — | Happy with current state | Sufficient time | No time/easier to eat unhealthily | — | Feeling hungry |
| — | No value | — | — | — | — |
