# Supplementary material for: Development and Testing of a Personalized Web-Based Diet and Physical Activity Intervention Based on Motivational Interviewing and the Self-Determination Theory: Protocol for the MyLifestyleCoach Randomized Controlled Trial
Source: JMIR Res Protoc. 2020 Feb 4;9(2):e14491. doi: 10.2196/14491 (PMC7055747; doi:10.2196/14491)
Supplement: Multimedia Appendix 2 [file resprot_v9i2e14491_app2.docx]

**Multimedia Appendix 2. Translation of the Motivational Interviewing (MI) spirit into our Web-based CT intervention.**

Here we describe how the spirit of MI, or “the set of heart and mind with which one enters into the practice of MI”, has been applied in the intervention, which is based on Friederichs and colleagues [1, 2]. Although it is challenging to apply certain elements (e.g. expressing genuine empathy and responding to subtle expressions of motivation such as change talk) in an online platform, Web-based, CT offers a way to reach many individuals and can respond to the user’s input by providing very specific feedback messages. Multimedia Appendix Table 2 presents an overview of the MI’s spirit, the definitions of these principles and how we applied these principles in the intervention.

Multimedia Appendix Table 2. An overview of the implementation of the spirit (partnership, acceptance, compassion, evocation) of MI in the intervention.

| **MI spirit** | **Definition** | **Application** |
| --- | --- | --- |
| Partnership | It concerns the collaboration between the therapist and the client. MI is done “for” and “with” a person and not “to” or “on” [2]. The MI counsellor explores the ideas of the client, shows interest for the client and supports him/her to create a positive interpersonal environment instead of being coercive or persuasive. The client decides whether he/she wants to change and their best way to achieve behavioral change. This approach facilitates the client’s basic psychological need for relatedness [3]. | - Description of participants’ view on several topics (e.g. importance of eating healthily)  - Participants reflect on statements they made earlier, which serves as an opening in each session or as input for feedback or used for a collaborative conversation.  - Participants choose whether they want to eat more healthily and/or become more physically active. The program does not give judgmental feedback on their choices.  Furthermore, the program gives tools to the participants but it is up to them how they “fill in” those tools (e.g. action plan). |
| Acceptance & compassion | An attitude of profound acceptance for the client, and a priority for the client’s needs. Acceptance involves respecting the person’s autonomy (which is a basic psychological need), their irrevocable right and capacity of self-direction. In line with acceptance lies compassion, which is a “deliberate commitment to pursue the welfare and best interests of the other” [2]. | - Participants are seen as experts of their own behavior and they decide themselves what and how they want to change (explicitly stated). There are many tailored specific feedback messages, written in an empathetic style, to connect to the client’s needs and wishes. |
| Evocation | By using evocation, the counsellor works to evoke the client’s reason for change and ideas how to achieve this change and stands in contrast to trying to convince the client to change [2]. | See Multimedia Appendix 3 and 4, how we tried to evoke the participant’s own motivation and confidence. |

**References**

1. Friederichs SAH, Oenema A, Bolman C, Guyaux J, van Keulen Hilde M, Lechner L. I Move: systematic development of a web-based computer tailored physical activity intervention, based on motivational interviewing and self-determination theory. BMC Public Health 2014 Feb 28; 14:212

2. Miller W, Rollnick S. Motivational Interviewing: Helping People Change, 3rd Edition. New York: The Guilford Press; 2013.

3. Markland D, Ryan RM, Tobin VJ, Rollnick S. Motivational Interviewing and Self–Determination Theory. J Soc Clin Psychol 2005 Sept; 24(6):811-831.
