# Supplementary material for: Development and Testing of a Personalized Web-Based Diet and Physical Activity Intervention Based on Motivational Interviewing and the Self-Determination Theory: Protocol for the MyLifestyleCoach Randomized Controlled Trial
Source: JMIR Res Protoc. 2020 Feb 4;9(2):e14491. doi: 10.2196/14491 (PMC7055747; doi:10.2196/14491)
Supplement: Multimedia Appendix 3 [file resprot_v9i2e14491_app3.docx]

**Multimedia Appendix 3. Translation of MI processes into our Web-based CT intervention.**

MI consists of four fundamental processes: engaging, focusing, evoking and planning. Multimedia Appendix Table 3 presents how we applied these processes in our Web-based CT intervention.

Multimedia Appendix Table 3. An overview of the implementation of processes of MI in the intervention.

| **MI process** | **Definition** | **Application** |
| --- | --- | --- |
| Engaging | Engaging is the process in which the client and counsellor establish a helpful connection and a working relationship [1]. It is assumed that this supports the basic psychological need for relatedness [2, 3]. | - At the start of the intervention, participants get information about the nature of the program. - At the beginning of each session, an overview of the content of the session is given. - To create a sort of social relationship with the program, we chose to include a video coach [4]. Our video-coach speaks to the participant, introduces and concludes each session. - The video-coach briefly introduce new questions and exercises (for example about motivation and confidence) and introduce former participants of the intervention. - We include narratives of former participants of the intervention, to facilitate the opportunity to feel connected with others with similar experiences, to enhance relatedness [2]. For all video applies that participants can choose whether they want to watch the videos, and which one(s) they want to watch. |
| Focusing | Focusing is the ongoing process of seeking and maintaining direction and within it more specific achievable goals [1]. | - Participants are told that the intervention is about eating more healthily and PA before the start of the intervention. - Participants are encouraged to come up with their own themes and ideas about eating healthily. In this way, we try to address the several sources from which it is possible to derive focus and direction, namely the participant (“client”) and program (“setting” and “clinical expertise”). - Participants can choose for themselves which module (*I Eat*, *I Move*, both or none) to follow in the opening session. |
| Evoking | Evoking is eliciting the client’s own motivation for change, which is also linked to the basic psychological need for autonomy and competence [1, 2]. These strategies are important to elicit change talk. | **Importance ruler:** rate the level of perceived importance on a scale [5]. For example (in the opening session, session 1 and 3), “How important would you say it is for you to eat more healthily on a scale ranging from 1 to 10?   - When the score is between 5 and 7, the following questions are presented: 1) It looks like eating more healthily is not totally unimportant to you, but neither is it totally important, why is eating (more) healthily not so important to you?’ and 2) ‘Why did you not choose a lower number?’. The aim of these questions is to identify barriers and motivational statements that could evoke change talk. - When the score is 8 or higher, the question ‘why is eating more healthily so important to you?’ is asked. The aim of this question is to foster motivational statements. - When the score is 4 or lower, this question follows: ‘It looks like eating more healthily is not on top of your list. Maybe you still have reasons why becoming eating more healthily could be important to you though. Why could eating more healthily be important to you?’ The aim of this question is to foster self-determined motivational statements. - Then the participants are asked to select an answer from a list of predefined categories that best reflects their answer (see pilot study 2). - After that, the participants receive a reflective feedback message of which the content depends on their importance ruler score and the answer(s) from the follow-up question(s). - For the participants with an average score (5-7), this feedback message mainly elaborates on the discrepancy between the barriers and motivational statements. - For the participants with a high score (8-10), the feedback message highlights the participants’ strong motivation, including their most important reason for having this strong motivation. - For the participants with a low score (1-4), the feedback message elaborates on the difference between the low importance score and the reason for why eating more healthily could be important for the participant. - We wrote 21 different feedback messages to optimally fit each individual’s answer. For example,   *“You certainly have reasons to eat more healthily, however, you think it so expensive. Many people think that healthy food is expensive, but healthy groceries do not have to be expensive and sometimes they may even be cheaper. Some vegetables and fruits are cheaper in a certain season. Choose especially these seasonal products. Frozen or unprepared vegetables, or vegetables from canned, pot or frozen are also often cheaper than pre-cut/prepared vegetables. Go to the store and look for and compare prices. Keep an eye on the offers for healthy products. If long-life products such as (wholegrain) rice or frozen vegetables are on offer, it is wise to buy them in larger quantities. Products like meat or fish are often the most expensive part of the meal. You could replace these once in a while with legumes. What helps many people is compare the costs of healthy versus unhealthy food. In this way, a better idea can be obtained on how important healthy food really is for them. Maybe this could also be an idea for you?*  **Exploring values:** explore what people care most about and what values they choose to guide their lives [1]. We ask the participant the open question: “What are your two most important values in life?”. After writing down two values, the participant is asked how eating more healthily may be linked to these values. Then the participant gets an overview of this exercise and is asked to think a little bit more on this topic.  **Looking forward:** the participant is asked to imagine that he or she would eat more healthily and that he or she would maintain this new behavior for a period of five years to boost the participants’ confidence. Then the participant is asked what positive effects this better dietary pattern would entail for him or her, i.e. how he/she would feel, and whether this influences how he/she thinks about eating more healthily.  **Confidence ruler:** rate the level of perceived confidence on a scale. For example, “How confident would you say you are to eat more healthily? On a scale ranging from 1 to 10, where 0 is not at all confident and 10 is extremely confident, where would you say you are?”. Depending on the given scores, the following questions to elicit the participants’ perspectives of confidence are presented:   - A given score between 5 and 7: 1) It looks like you have some confidence, but you are not totally confident, why did you not choose a higher number?’ and 2) ‘Why did you not choose a lower number?’. The aim of these questions is to identify barriers to confidence and drivers/personal strengths that could boost confidence. - A given score of 8 or higher: ‘why are you so confident that you could eat more healthily?’. The aim of this question is to identify drivers or personal strengths of confidence. - A given score 4 or lower: ‘It looks like you are not very confident. What would help you to gain more confidence?’. The aim of this question is to stimulate the participants to think about ways to increase their confidence.   Then the participants are asked to select an answer from a list of predefined categories that best reflects his/her answer (see pilot study2). After that, the participants receive a reflective feedback message of which the content depends on their confidence ruler score and the answer(s) from the follow-up question(s). If the score is:  - between 5 and 7: the feedback message is mainly composed of the barriers and the facilitators of confidence.  - 8 or higher: the feedback message highlights the participants’ strong confidence and personal strengths.  - 4 or lower, the feedback message mainly addresses how the participant could become more confident.  For I *Eat*, we wrote 140 unique feedback messages that optimally fit each individual’s answer. This is an example of such a feedback message:  *You have difficulty resisting temptations. As a result, you are less confident that you will be able to eat more healthily. That is very understandable! You are a real go-getter; if you want to do something, then you are going for it all the way. Despite the fact that you find it difficult to resist temptation, you, fortunately, have some confidence that you would be able to eat more healthily. You also said the following about this: “Once I want something, I’m unstoppable”.*  *A good plan to deal with temptations can help with eating more healthily. Creating an action plan will come back later in this session. Do you already have any ideas for this plan? For example, how you could resist those temptations? Then remember this for later.*  **Looking back:** the client recalls a moment in the past before the problem emerged to emphasize the discrepancy between the past and present with the possibility of life being better again. For example: ‘Please think back to a moment in time when you ultimately succeeded in doing something very difficult. What did you have to do? How did you ultimately succeed? How did you feel when you had succeeded? This is followed by an overview of the participants’ answers. Then we ask: “Could looking back on this experience help you in becoming more confident that you also could succeed in eating more healthily?’ |
| Planning | Planning is the process in which a person thinks about how and when to change. This happens when a threshold of readiness is reached [1]. This process consists both of developing commitment to change and formulating a specific action plan. | **Action plan**: Participants are given the opportunity to make a specific action plan at the end of the session. In *I Eat*, we slightly adjusted the different aspects of the action plan.   - Participants start by indicating how they want to eat more healthily, then they state their most important motivational reasons for eating more healthily, where and when they want to do it, when they would like to start with eating more healthily, whether and which preparations they have to do in order to start eating more healthily and how they notice whether the plan is working. Participants are also asked with whom they want to eat more healthily. - Participants who decide to not make an action plan in the first session, get the possibility again in the following sessions to create an action plan. This option is specifically implemented for the ones have not reached their readiness potential. - When a participant has made an action plan, the execution of this plan is evaluated in the subsequent session. They are encouraged to think about factors/situations that supported or hindered them in executing their plan. Participants are also able to adjust their plan in each of the follow-up sessions.   **Coping plan**: Participants can also create coping plans, in which they can write down barriers that may hinder performing the planned behavior, such as difficult situations, and how they can overcome these barriers [6-9]. Such coping plans are based on an *if-component*, which is the barrier/difficult situation and a *then-component*, which is the coping strategy of how to deal with the barriers [7]. We implemented coping plans by first asking the participant to describe two difficult situations in which it would be hard to execute his/her action plan. This is followed by a question asking how the participant could cope with these difficult situations. Lastly, the participant gets a summary of their coping plan. |

**References**

1. Miller W, Rollnick S. Motivational Interviewing: Helping People Change, 3rd Edition. New York: The Guilford Press; 2013.

2. Markland D, Ryan RM, Tobin VJ, Rollnick S. Motivational Interviewing and Self–Determination Theory. J Soc Clin Psychol 2005 Sept; 24(6):811-831.

3. Ryan RM, Lynch MF, Vansteenkiste M, Deci EL. Motivation and autonomy in counseling, psychotherapy, and behavior change: A look at theory and practice. Couns Psychol 2011;39(2):193-260.

4. Friederichs S, Bolman C, Oenema A, Guyaux J, Lechner L. Motivational interviewing in a Web-based physical activity intervention with an avatar: randomized controlled trial. J Med Internet Res. 2014 Feb;16:e48.

5. Rollnick S, Butler CC, Stott N. Helping smokers make decisions: the enhancement of brief intervention for general medical practice. Patient Educ Couns 1997 Jul; 31(3):191-203

6. Allan JL, Sniehotta FF, Johnston M. The best laid plans: Planning skill determines the effectiveness of action plans and implementation intentions. Ann Behav Med. 2013 Aug;46:114-120.

7. Scholz U, Schüz B, Ziegelmann JR, Lippke S, Schwarzer R. Beyond behavioural intentions: Planning mediates between intentions and physical activity. Br J Health Psychol. 2008 Sept;13:479-494.

8. Sniehotta FF. Towards a theory of intentional behaviour change: plans, planning, and self-regulation. Br J Health Psychol. 2009 May;14(Pt 2):261-273.
