# Supplementary material for: Development and Testing of a Personalized Web-Based Diet and Physical Activity Intervention Based on Motivational Interviewing and the Self-Determination Theory: Protocol for the MyLifestyleCoach Randomized Controlled Trial
Source: JMIR Res Protoc. 2020 Feb 4;9(2):e14491. doi: 10.2196/14491 (PMC7055747; doi:10.2196/14491)
Supplement: Multimedia Appendix 4 [file resprot_v9i2e14491_app4.docx]

**Multimedia Appendix 4: Translation of core MI skills into our Web-based CT intervention**

There are several communication skills that are important in MI. These core skills are asking open questions, affirming, reflecting, summarizing and informing/advising. Multimedia Appendix Table 4 presents the translation of these core communication skills into our Web-based CT intervention.

Multimedia Appendix Table 3. An overview of the implementation of the core skills of MI in the intervention.

| **MI skills** | **Definition** | **Application** |
| --- | --- | --- |
| Open questions & reflective listening | By asking *open questions*, a person is invited to think before responding and encourages a person to a full, meaningful answer or insights and reasons for change [1, 2]. This supports autonomy (SDT) more than having a limited set of response options [3].  In a *reflective statement*, a guess is made what the client means to get a deeper understanding by clarifying whether one’s guess is correct [1]. It is important to engage others in a relationship, build trust and foster motivation. | We used a structured approach to “interpret” and “react” to the user’s input compared to a human counsellor, in which participants answer an open question and then are asked select in a list of predefined answers the option that resembles their answer best. We wrote a unique feedback message for each combination of the multiple-choice answers (i.e. the categories). In these messages, we repeated the selected answer and tried to add some extra meaning to all messages to create a sort of understanding (“skillfulness”). |
| Affirming | *Affirming* is the skill in which counsellor responds to the client’s personal strengths, efforts and resources that lead in the direction of behavioral change [1]. In the context of SDT, affirming supports the basic psychological needs for competence and relatedness [4]. | We use an empathic style in the program and feedback messages to simulate affirming. For example, if a participant only improves on one domain in their diet, then the feedback is positive for that domain and stating that it is hard to change a diet completely. Another example is that when a participant does not succeed in eating more healthily, the attempt for trying is rewarded, and emphasizes that a failed attempt does not mean that the participant is not able to change. |
| Summarizing | *Summaries* are applications of reflections in which the main points that the client has told are offered, helping to reflect on the various experiences that the client addresses [1]; this fosters the client’s need for autonomy by hearing his or her own ideas/thoughts back from the counsellor; and competence by structuring the conversation [4]. | Participants regularly receive summaries and specifically at transition points, e.g. at the end of an exercise such as value exploration.  A day after the participants have finished a session, they receive an email containing a pdf file in which a summary of the topics discussed and their answers from that particular session are stated. |
| Informing & advising | Informing and advising are appropriate in MI as long as it is offered with permission or as it serves as a way to understand the client’s perspective and needs, and to help the client to reach his/her own conclusions about any information provided by the counsellor, thus without interpreting this information any further [1]. This supports the basic psychological need “competence” [4]. | Participants are introduced to the guidelines about a healthy diet and if they wish they can get more information about these topics.  Furthermore, there are several short videos in which an expert tells something about the benefits of a healthy diet on several domains such as health or physical appearance. Participants can decide for themselves whether they want to see these videos. |

**References**

1. Miller W, Rollnick S. Motivational Interviewing: Helping People Change, 3rd Edition. New York: The Guilford Press; 2013.

2. Patrick H, Williams GC. Self-determination theory: its application to health behavior and complementarity with motivational interviewing. Int J Behav Nutr Phys Act 2012 Mar 02; 9:18

3. Ryan RM, Lynch MF, Vansteenkiste M, Deci EL. Motivation and autonomy in counseling, psychotherapy, and behavior change: A look at theory and practice. Couns Psychol. 2011 Feb;39(2):193-260.

4. Markland D, Ryan RM, Tobin VJ, Rollnick S. Motivational Interviewing and Self–Determination Theory. J Soc Clin Psychol 2005 Sept; 24(6):811-831.
